# Spatial–Temporal Self-Attention Enhanced Graph Convolutional Networks for Fitness Yoga Action Recognition

**DOI:** 10.3390/s23104741

**Published:** 2023-05-14

**Authors:** Guixiang Wei, Huijian Zhou, Liping Zhang, Jianji Wang

**Affiliations:** 1 School of Sports Center, Xi’an Jiaotong University, Xi’an 710000, China; wgx.627@mail.xjtu.edu.cn (G.W.);; 2 School of Software Engineering, Xi’an Jiaotong University, Xi’an 710000, China; zhj11055331@stu.xjtu.edu.cn; 3 Institute of Artificial Intelligence and Robotics, Xi’an Jiaotong University, Xi’an 710000, China

**Keywords:** fitness yoga, human action recognition, self-attention mechanism

## Abstract

Fitness yoga is now a popular form of national fitness and sportive physical therapy. At present, Microsoft Kinect, a depth sensor, and other applications are widely used to monitor and guide yoga performance, but they are inconvenient to use and still a little expensive. To solve these problems, we propose spatial–temporal self-attention enhanced graph convolutional networks (STSAE-GCNs) that can analyze RGB yoga video data captured by cameras or smartphones. In the STSAE-GCN, we build a spatial–temporal self-attention module (STSAM), which can effectively enhance the spatial–temporal expression ability of the model and improve the performance of the proposed model. The STSAM has the characteristics of plug-and-play so that it can be applied in other skeleton-based action recognition methods and improve their performance. To prove the effectiveness of the proposed model in recognizing fitness yoga actions, we collected 960 fitness yoga action video clips in 10 action classes and built the dataset Yoga10. The recognition accuracy of the model on Yoga10 achieves 93.83%, outperforming the state-of-the-art methods, which proves that this model can better recognize fitness yoga actions and help students learn fitness yoga independently.

## 1. Introduction

Human action recognition is a hot research issue in the field of computer vision, which is the main method to help learn and understand human action. Data obtained by cameras or 3D sensors can be recognized to analyze human actions and preserve action information. Human action recognition can be applied in many applications, such as biometric recognition, video monitoring, assisted living, sports arbitration, and intelligent human–computer interaction [1,2,3]. Human action recognition technology is also applied in yoga practice to improve the accuracy of yoga actions. Researchers have proposed many different models to guarantee the accuracy of action recognition. Human action recognition needs to be recognized by obtaining different data modalities, such as RGB [4,5,6,7], optical flow [8,9], skeleton [10,11,12,13], and so on. In this paper, we try to establish a skeleton-based model on the basis of yoga video clips to identify the standards of yoga actions.

Compared with other types of models, the skeleton-based model of human action recognition has the following advantages. First and foremost, skeleton data do not contain information of human appearance or scenes where people perform the actions. Therefore, the skeleton-based models will not be influenced by occlusion and illumination changes and can accurately reflect the real types of one action. In addition, as the size of skeleton data is smaller than that of RGB video data, skeleton-based methods generally need less computation and less memory space.

When students learn yoga independently, they may learn in different scenes, such as school, gym, home, park, and so on. This will lead to more complex and diverse background information, making it difficult to classify the fitness yoga actions. However, because the skeleton data do not contain the background information of the learning scene, the skeleton-based human action recognition can overcome the difficulty of complex background information. At the same time, as students need to obtain feedback to judge whether their actions are correct at the time when learning fitness yoga, the algorithm is required to be highly real-time. As mentioned earlier, skeleton-based human action recognition needs less computation compared with methods based on other modalities. In this way, the model can obtain faster computation speed and meets real-time requirements.

Generally speaking, skeleton data can be obtained in the following two ways:

(1) Given the RGB videos, the 2D coordinates of human joints in the video frames are estimated by the pose estimation algorithms to obtain the human skeleton data. RGB videos can be collected from video websites or RGB cameras.
(2) The 3D coordinates of human joints can be directly captured by the depth sensors, so as to obtain the human skeleton data.

Since one of the application scenarios of this research is that students learn fitness yoga independently, videos captured by mobile phones are used to judge the accuracy of fitness yoga actions. So, we use this method to obtain the skeleton data as the input data of the model proposed in this paper.

Because human action based on the skeleton can be naturally represented by a chronological series of graphs, which consist of human joint locations that can be represented as 2D or 3D coordinates as points and natural connections between human joints as edges, Yan et al. applied GCNs to model the dynamic human skeleton [14], and proposed the spatial temporal graph convolutional networks (ST-GCNs). The ST-GCN can automatically capture spatial and temporal features by applying GCNs to skeleton-based action recognition tasks without hand-crafted parts, which also leads to higher performance and better expressive power than previous work based on temporal CNNs [13,15] or RNNs [16,17]. Therefore, GCNs are usually used as the backbone of skeleton-based action recognition and we also use GCNs in the present research.

However, as a pioneering work of applying GCNs to the task, ST-GCN also has many drawbacks to be improved. In the past few years, researchers have improved models by constructing more flexible graph topology [11,12,18], applying multi-stream input [19,20], and representing skeletons using heatmaps [21]. Among these improvements, we notice that there is still room for improvement in the attention mechanism of the model. Inspired by the successful application of self-attention mechanisms in many areas, such as natural language processing [22], image segmentation [23], and object detection [24], we try to perfect the model from the perspective of the self-attention mechanism.

To sum up, in order to better recognize fitness yoga actions, we propose a new skeleton-based action recognition method—spatial–temporal self-attention enhanced graph convolutional networks (STSAE-GCNs). In this method, the spatial–temporal self-attention module (STSAM) is applied to improve the spatial–temporal expression ability of the model, and thus to better recognize fitness yoga actions. To verify the recognition ability of the model for fitness yoga actions, we collected 960 short videos (about 3 s) of college students’ yoga actions and established the dataset Yoga10.

The major contributions of this paper lie in three points:

(1) A new skeleton-based action recognition method for fitness yoga, the spatial–temporal self-attention enhanced graph convolutional network (STSAE-GCN) is proposed to better recognize fitness yoga actions.
(2) The spatial–temporal self-attention module (STSAM) that can improve the spatial–temporal expression ability of the model is presented. The STSAM has the characteristics of plug-and-play and can be applied in other skeleton-based action recognition methods.
(3) A dataset Yoga10 of 960 videos is built. The STSAE-GCN proposed in this research achieves 93.83% recognition accuracy on Yoga10, and outperforms state-of-the-art methods. The Yoga10 dataset can provide a unified verification basis for future fitness yoga action recognition.

## 2. Related Work

### 2.1. Skeleton-Based Action Recognition

Since skeleton-based action recognition is robust to illumination change and occlusion problems with less computation and storage compared with other modalities-based methods [4,5,8,9], researchers proposed many skeleton-based methods for action recognition. As the human skeleton is a good graph structure with joints as points and bones as edges, Yan et al. proposed spatial–temporal graph convolutional networks (ST-GCNs) by first applying GCNs to a skeleton-based action recognition task [14], which have achieved better performance compared with previous RNN-based methods [16,17] and temporal CNN-based methods [13,15]. Recently, many models have been proposed to improve the performance of GCN-based action recognition. Li et al. constructed a more flexible graph topology to establish graph connections between disconnected skeleton joints [11]. Shi et al. applied bone information and motion information to construct a multi-stream input for the model [19]. Chen et al. expanded the graph topology to the channel level so that the graph topology of each feature channel is different [18]. Malik et al. [25] proposed an HAR system with a fine-KNN classifier and an extraneous frame scrapping technique to overcome dimensionality problems. In order to better study the skeleton-based action recognition methods, Duan et al. have performed some good experiments using GCN-based methods and proposed the model ST-GCN++ [26], which is used as a baseline model in this research.

### 2.2. Attention Mechanism

Attention mechanism is actually a method to focus on more valuable task resource allocation in the case of limited computing resources. Among the many methods of attention mechanism, self-attention is one of the most excellent ones. Since it was proposed, it has been well applied in many areas, such as natural language processing [22], image segmentation [23], and object detection [24]. Inspired by these successful applications of self-attention, we attempt to apply self-attention in the skeleton-based action recognition task.

### 2.3. Yoga Pose Detection

In past decade, with the development of vision and sensor technology, many works have been performed on yoga pose detection [27]. In order to help people learn yoga independently, researchers have developed many methods to aid people in self-training and avoiding injury. Eyes-Free Yoga is an exergame that helps people who are blind or have low vision exercise to keep healthy [28]. Yao et al. [29] proposed an STF-ResNet that can better extract spatial–temporal correlation information to recognize yoga actions. By using Microsoft Kinect (a depth sensor tracks skeleton joints), Eyes-Free Yoga acts as yoga instructor to correct yoga actions and has personalized auditory feedback. Chen et al. also proposed a self-training system to assist in rectifying yoga postures [30]. The system extracts body contour, skeleton, dominant axes, and feature points to analyze the participants’ yoga postures by using Kinect to acquire data. In 2018, Trejo et al. proposed an interactive yoga postures recognition system that can track up to 6 people at the same time by using Kinect [31]. Other researchers also use Kinect to track the yoga postures [32,33,34]. However, it is expensive for people like students to buy an additional depth sensor. At the same time, it is also inconvenient for students to carry and is difficult to operate. So, in this research, we propose a yoga action recognition method which can use video captured by a mobile phone as the input data. In this way, students and other people can learn yoga more conveniently.

## 3. Method

**Figure 1:**
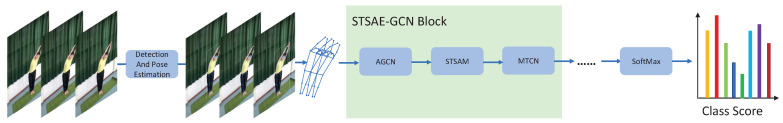
The framework of STSAE-GCN. The pose estimation is firstly performed on raw RGB videos to acquire the skeleton representation of actions. Nine layers of STSAE-GCNs will be applied to extract high-level features of actions. Finally, a standard softmax will be applied to generate the score of every action class. Different colors in the class score represent probability of different fitness yoga actions.

In order to judge whether the students’ actions are standard when they perform fitness yoga privately, we propose spatial–temporal self-attention enhanced graph convolutional networks (STSAE-GCNs), which can better recognize the actions of fitness yoga compared with the baseline model, ST-GCN++ [26]. The framework of the proposed model is shown in Figure 1. Usually, the skeleton data can be obtained by motion capture devices or pose estimation algorithms from videos. For this work, we obtain the skeleton using a pose estimation algorithm, HRNet [35], which firstly detects the position of the human in the video and then estimates the location of joints. Using pose estimation, a chronological series of the human skeleton will be obtained. Nine layers of STSAE-GCN will be applied to extract high-level features of actions. The features will go through an average pooling layer and a full connection layer whose number of channels is equal to the number of action categories. Finally, a standard softmax will be applied to generate the score of every action category. The SASAE-GCN block consists of three modules: an adaptive graph convolutional network (AGCN), a multi-branch temporal convolutional network (MTCN), and a spatial–temporal self-attention module (STSAM). The three modules will be introduced in the following subsections.

### 3.1. Adaptive Graph Convolutional Networks

By setting human joint locations as points and natural connections between human joints as edges, human body actions can be naturally represented by a chronological series of graphs. Moreover, in view of the advantages of graph convolutional networks (GCNs) in extracting the features of graphs, we introduce a GCN to extract the spatial features of skeleton data, which can be formulated as

(1) $$f_{out}=D^{-\frac{1}{2}}\left(A+I\right)D^{\frac{1}{2}}f_{in}W,$$

where $f_{in}$ represents the input features and $f_{out}$ represents the output features. The *A* adjacency matrix represents the intra-body connections of joints within a single frame and the identity matrix *I* represents the self-connections. *W* represents the weight matrix. *D* represents the degree matrix. In practice, a 1 × 1 2D convolution is applied to the input features $f_{in}$. The output features of 2D convolution will be fused according to the adjacency matrix *A*. Then, we will obtain the output features $f_{out}$ of GCN. Another point that needs to be paid attention to is that the adjacency matrix *A* is not fixed. For example, in the clapping action, the two hand joints have a strong correlation, but in the natural connection of the human body, the two hand joints are not directly connected. As a result, if the adjacency matrix *A* is fixed as the natural connection of the human skeleton, the ability of the GCN to extract spatial features will decline. Therefore, in order to enable the GCN to better extract the spatial features of the skeleton data, we initialize the adjacency matrix *A* with the natural connection of the human skeleton and make the adjacency matrix *A* learnable. In this way, we can obtain a better adjacency matrix *A* that is adaptive to the data. Finally, we obtain an adaptive graph convolutional network (AGCN), which can better extract the spatial features of the skeleton.

### 3.2. Multi-Branch Temporal Convolutional Networks

**Figure 2:**
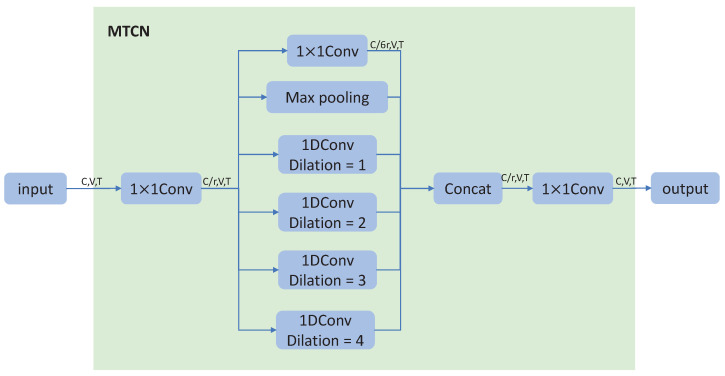
The MTCN. Given the input, an 1 × 1 convolution operation is first applied to reduce the number of channels, which can greatly reduce the computation requirements. Then, the features will go through six branches. The number of output features channels per branch is one-sixth of the input features. The output of six branches will be concatenated together and go through another 1 × 1 convolution operation.

An action includes not only the spatial features of the relative positions of joints but also the temporal features of joints’ motion in adjacent frames. An AGCN is applied to extract spatial features, while a TCN is applied to extract temporal ones. Unlike the single temporal branch used in most GCN-based methods [11,14], inspired by [18,20], we applied multi-branch temporal convolutional networks (MTCNs) to replace the single branch. The structure of an MTCN is shown in Figure 2. There are six branches in total: a ‘1 × 1’ Conv branch, a Max-Pooling branch, and four temporal 1D Conv branches with kernel size 3 and dilations from 1 to 4. Given the input, an 1 × 1 convolution operation is first applied to reduce the number of channels, which can greatly reduce the computation. Then, the features will go through six branches, which will focus on the temporal features of different scales, respectively. At the same time, in order to avoid excessive calculation, the number of output features channels per branch is one-sixth of the input features. Finally, the output of six branches will be concatenated together and go through another 1 × 1 convolution operation. Compared to single branch of a TCN, the MTCN will not only lead to better performance, but also save the computational cost and parameters.

### 3.3. Spatial–Temporal Self-Attention Module

**Figure 3:**
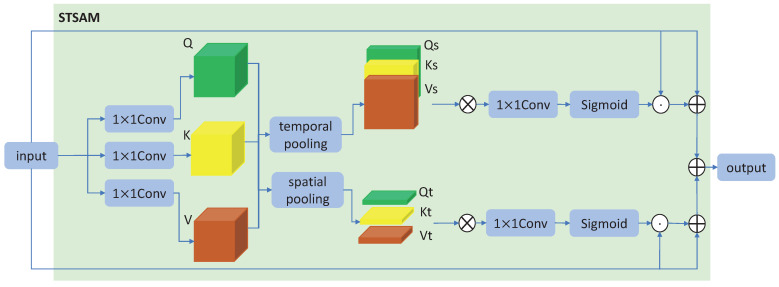
The STSAM. Given the input, three 1 × 1 convolution operations are applied to obtain *Q*, *K*, *V*. Then, the pooling operations of T and V dimensions are, respectively, applied to Q,K,V to obtain the $Q_{s},K_{s},V_{s}$ and $Q_{t},K_{t},V_{t}$. The features will go through the ⊗ operation, 1 × 1 convolution operation, and sigmoid operation to obtain the final spatial attention map and temporal attention map. ⊗ represents Equations (2) and (3), ⊙ represents element-wise multiply, and ⊕ represents element-wise add.

The attention mechanism is applied in many skeleton-based action recognition methods [19,36] to achieve better performance. Inspired by the successful application of the self-attention mechanism in natural language processing tasks [22], we propose a spatial–temporal self-attention module (STSAM) to apply the self-attention mechanism to skeleton-based action recognition. The detailed structure of STSAM is shown in Figure 3.

Given the input $F_{in}\in R^{C\times T\times V}$, three 1 × 1 convolution operations are, respectively, applied to obtain *Q* (query), *K* (key), and *V* (value). For dimensions of input data, *C* is the number of channels, *T* is the number of frames, and *V* is the number of joints. Secondly, in order to obtain the spatial attention map and temporal attention map of skeleton features, respectively, the pooling operations of *T* and *V* dimensions are, respectively, applied to $Q,K,V\in R^{C\times T\times V}$. Then, the $Q_{s},K_{s},V_{s}\in R^{C\times 1\times V}$ and $Q_{t},K_{t},V_{t}\in R^{C\times T\times 1}$ are obtained. To compute the attention map, the following Equations (2) and (3) are applied to $Q_{s},K_{s},V_{s}$ and $Q_{t},K_{t},V_{t}$, respectively, which can be represented as

(2) $$M_{s}=softmax\left(\frac{Q_{s}K_{s}^{T}}{\sqrt{d_{k}}}\right)V_{s},$$

(3) $$M_{t}=softmax\left(\frac{Q_{t}K_{t}^{T}}{\sqrt{d_{k}}}\right)V_{t},$$

where $\sqrt{d_{k}}$ is the number of channels, $M_{s}\in R^{C\times 1\times V}$ represents the spatial attention map, and $M_{t}\in R^{C\times T\times 1}$ represents the temporal attention map. To guarantee more stability of the performance of the model, the number of channels of the attention map will be scaled to 1 by another 1 × 1 convolution operation. Finally, the activation function sigmoid is applied to obtain the final attention map. The two steps can be formulated as

(4) $$M_{s_{1}}=\delta \left(W_{s}*M_{s}\right),$$

(5) $$M_{t_{1}}=\delta \left(W_{t}*M_{t}\right),$$

where δ represents the sigmoid operation, and $W_{s}$ and $W_{t}$ represent the 2D convolution layer with kernel size 1 × 1. Finally, we add the spatial attention map and temporal attention map into the model using the residual blocks, which can be formulated as

(6) $$F_{out}=\left(F_{in}+F_{in}⊙M_{s_{1}}\right)+\left(F_{in}+F_{in}⊙M_{t_{1}}\right),$$

where $F_{out}\in R^{C\times T\times V}$ represents the output features, and ⊙ represents the element-wise multiply operation.

## 4. Experiments and Discussion

In this section, we evaluate the performance of the STSAE-GCN in skeleton-based action recognition experiments. We experiment on the dataset Yoga10 that we have collected for this work. All experiments were conducted on PyTorch deep learning framework.

### 4.1. Dataset

Yoga10. Yoga10 is the dataset collected for this work. In total, it has 960 video clips in 10 action categories. The actions are performed by 32 volunteers indoors with 3 cameras to record the videos at different angles at the same time. The 10 action categories are (1) Wind-Blown Tree Pose; (2) Skyscraper Pose; (3) Straight Angle Pose; (4) Moon Pose; (5) Warrior 2 Pose; (6) Chair Pose; (7) Locust Pose; (8) Plank Pose; (9) Downward-Facing Dog Pose; (10) Half Boat Pose. The dataset is divided into 150 and 810 clips for training and evaluation. The training clips come from one subset of volunteers and the models are evaluated on clips from the remaining actors. Because the Yoga10 dataset only includes raw RGB videos without skeleton data, we use a top-down pose estimation algorithm, HRNet [35] pre-trained on COCO-keypoint [37], to obtain the 2D poses. The obtained skeleton has 18 joints and every joint is represented by a 2D coordinate and the confidence of the joint. We evaluate the recognition performance by top-1 and top-5 classification accuracy. Top-1 accuracy refers to the accuracy of the first ranked category matching the actual results. Top-5 accuracy refers to the accuracy of the top five categories containing the actual results.

### 4.2. Ablation Study

In this section, we will evaluate the effectiveness of the proposed modules. We use ST-GCN++ [26] as a baseline for the experiments. The ST-GCN++ is the model that applies many good practices used in GCN-based approaches. It is the STSAE-GCN without the STSAM. We first evaluate the effectiveness of STSAM and the results are shown in Table 1. In fact, STSAM can be divided into two parts, spatial self-attention module (SSAM) and temporal self-attention module (TSAM). We added these two modules into the baseline model, respectively, as the models “SSAM” and “TSAM”. Then, the two modules are combined together as the model “STSAM”. To reduce the error of the experiments, we conduct every model setting five times and compute the average results of five experiments. From Table 1, the models with the best performance, applying the proposed modules, are consistently better than the baseline model. The average performance of the proposed models is also better than the baseline model. The model with two self-attention modules achieves the best performance.

The spatial self-attention module and temporal self-attention module are placed in parallel in the model “STSAM”. However, the two modules can also be placed in series, and the series can be placed in different orders. So, in order to determine which placement strategies can make the model achieve the best performance, the experiments are conducted here. The results of the experiments are shown in Table 2. The model setting “S-T” refers to the model in which features go through the spatial self-attention module first, and then go through the temporal self-attention module. The model setting “T-S” refers to the model in which features go through the temporal self-attention module first, and then go through the spatial self-attention module. We can see from Table 2 that the model with two self-attention modules placed in parallel achieves the best performance.

Based on the above experiments, we obtain the best model setting “TSAM” for the final model of the STSAE-GCN. The STSAE-GCN achieves 93.83% top-1 classification accuracy of action recognition.

### 4.3. Comparison with State-of-the-Art Methods

We compare our STSAE-GCN with previous state-of-the-art methods on Yoga10. Every model is still evaluated five times in top-1 classification accuracy. The results are shown in Table 3.

We can see from Table 3, whether it is the average recognition accuracy or the highest recognition accuracy of five experiments, our proposed model outperforms the other state-of-the-art methods.

### 4.4. Plug-and-Play Spatial–Temporal Self-Attention Module

As an effective attention module, STSAM has the characteristics of plug-and-play for GCN-based action recognition. To verify this characteristic, we apply the STSAM in other GCN-based methods: AAGCN, MSG3D, and CTRGCN. Because GCN-based recognition methods are based on the skeleton, the implementation details are different. Most of them use graph convolutional networks in a single frame to extract spatial features first, and then use temporal convolutional networks to extract temporal features. So, we add the STSAM between the two modules as in the STSAE-GCN method. The results are shown in Table 4. A + means that the model applies the STSAM.

We can see from Table 4 that all the performances of the three models are improved by applying the STSAM with the CTRGCN being improved by 2.22%, AAGCN improves by 0.93%, and MSGCD improves by 1.11%. So, to further improve the performance of existing GCN-based action recognition methods based on the skeleton, applying the STSAM may be a good choice.

### 4.5. Discussion

In Yoga10, every fitness action is captured in three different views at the same time. In order to enhance the generalization ability of the model for perspective transformation when recognizing actions, we use three different views of the same action to train the model. However, in practice, in order to improve the recognition accuracy of the model, we naturally want the human body to perform yoga actions from the view of the highest recognition accuracy. So, we divide the test set of Yoga10 into 3 parts according to the view of video clips, where every part contains 270 video clips. The test results are shown in Table 5.

**Figure 4:**
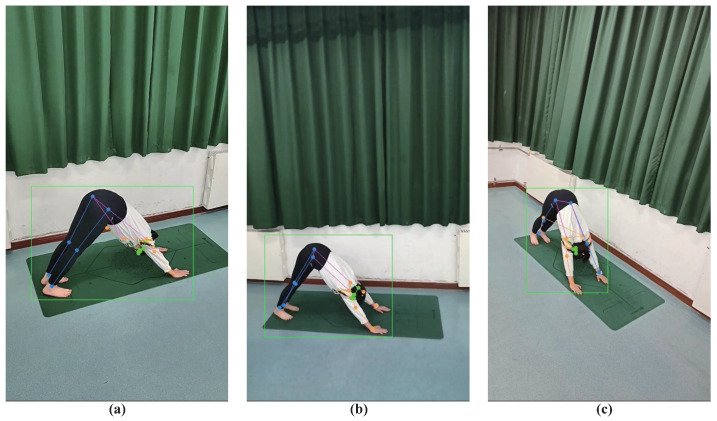
Fitness yoga action of Downward-Facing Dog Pose captured in three different views. (**a**) view-1. (**b**) view-2. (**c**) view-3. The blue dots represent the joints of the legs, the orange dots represent the joints of the arms, the green dots represent the joints of the head, and the green squares represent the human range calibrated by the human detection algorithms.

It can bee seen in Table 5 that view-2 achieves the best performance. Compared with the first two views, the recognition accuracy of view-3 is greatly reduced, which can be caused by the inaccurate pose estimation of view-3. Figure 4 is the result of pose estimation of Downward-Facing Dog Pose from three views.

In Figure 4, the pose estimation results of the first two views are relatively accurate, but in view-3, the arm joints are mapped to the legs, and the leg joints are mapped to the arms. As a result, the recognition accuracy of view-3 is lower than that of view-1 and 2. In this case, the recognition accuracy of the model shall be affected by the result of pose estimation. Therefore, in order to improve recognition accuracy, students should learn yoga independently from view-2. In a professional yoga competition or in the case of a conditional use of depth sensors, the influence of inaccurate pose estimation can be eliminated, so that the model can present a better recognition accuracy.

There are also occlusion problems. Many yoga actions have serious occlusion problems, which can lead to poor pose estimation results, as shown in Figure 4c. However, in most cases, even though there are minor occlusion problems, pose estimation algorithms can still accurately predict the position of skeleton points. Moreover, every video has many frames, and there are always frames that can find the correct position of the skeleton points. Finally, for the same yoga actions, they often have the same occlusion problems in the same views, which makes the predicted skeleton points still have a similar distribution, making our recognition algorithm still able to recognize the action well. Based on the above reasons, our model is robust to occlusion problems, which leads to the best recognition accuracy shown in Table 3.

### 4.6. Practical Application of Model

To explore whether the model can meet real-time requirements in future practical applications, we run our model on the NVIDIA GeForce RTX 2080 Ti. It costs 18.61 s to recognize 810 test skeleton clips in total. The average recognition time for each skeleton clip is only 0.02 s, which meets the requirements of real-time performance.

**Figure 5:**
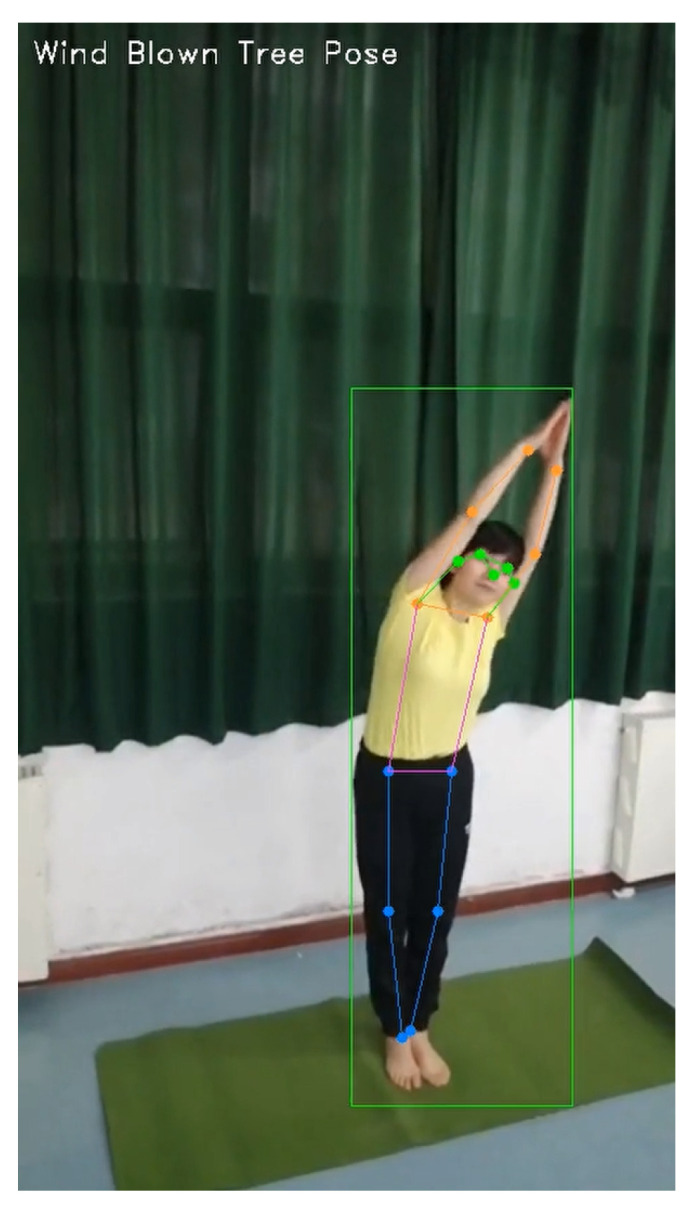
Wind-Blown Tree Pose. The text in the upper left corner represents the recognized action category. The blue dots represent the joints of the legs, the orange dots represent the joints of the arms, the green dots represent the joints of the head, and the green squares represent the human range calibrated by the human detection algorithms.

In practical applications, we will display the currently recognized actions of the model in the upper left corner of the video as shown in Figure 5. If the action recognized by the model is not the student’s current action, there is a high probability that the student’s action is incorrect and needs to be corrected.

## 5. Conclusions

In this paper, we propose a new skeleton-based action recognition method for fitness yoga action recognition, the spatial–temporal self-attention enhanced graph convolutional network (STSAE-GCN). This method greatly strengthens the spatial–temporal expression of the model, especially enhancing the performance by the spatial–temporal self-attention module (STSAM). As an attention module, STSAM has the characteristics of plug-and-play. It can be applied to other skeleton-based action recognition methods to further improve their performance of action recognition. We build the Yoga10 dataset to prove the performance of the model. STSAE-GCN achieved 93.83% recognition accuracy on Yoga10, outperforming the state-of-the-art methods. This method can be used as the most effective and intuitive scientific auxiliary means to assist students to learn fitness yoga independently or unify the judgment rules of fitness yoga competitions, which as a whole further promote the development of fitness yoga.

## Figures and Tables

**Table 1:** The ablation study of STSAM on Yoga10. Every setting is evaluated 5 times using top-1 classification accuracy. The average results of five experiments are also computed. The meaning of every setting please refers to Section 4.2.

Model	1	2	3	4	5	Average
baseline	89.51	87.65	90.49	90.62	90.99	89.85
SSAM	88.15	92.47	91.85	92.10	91.73	91.26
TSAM	91.48	92.59	90.99	90.49	89.14	90.94
STSAM	93.58	93.46	92.47	93.83	92.22	93.11

**Table 2:** The experiments on the placement of two self-attention modules on Yoga10. Every setting is evaluated 5 times by top-1 classification accuracy. Average results of five experiments are also computed.

Model	1	2	3	4	5	Average
S-T	91.48	92.84	87.53	92.72	90.62	91.04
T-S	88.89	91.23	91.98	89.88	92.10	90.82
STSAM	93.58	93.46	92.47	93.83	92.22	93.11

**Table 3:** Comparison with state of the art methods on Yoga10. Models are evaluated 5 times by top-1 classification accuracy. Average results of five experiments are also computed.

Model	1	2	3	4	5	Average
AAGCN [19]	92.10	90.37	88.27	88.40	86.30	89.09
MSG3D [20]	91.60	90.62	92.22	90.62	92.22	91.46
CTRGCN [18]	84.69	87.78	87.28	90.25	89.51	87.90
ST-GCN++ [26]	89.51	87.65	90.49	90.62	90.99	89.85
STSAE-GCN(ours)	93.58	93.46	92.47	93.83	92.22	93.11

**Table 4:** Verification of the plug-and-play characteristic of STSAM on Yoga10. Models are evaluated by top-1 and top-5 classification accuracy. + means that the model applies the STSAM.

Model	Top-1	Top-5
CTRGCN [18]	90.25	98.89
CTRGCN+	92.47	99.38
AAGCN [19]	92.10	98.77
AAGCN+	93.03	99.26
MSG3D [20]	92.22	99.26
MSG3D+	93.33	99.38

**Table 5:** The recognition ability of the model from different views. Models are evaluated by top-1 and top-5 classification accuracy.

View	Top-1	Top-5
view-1	96.30	99.63
view-2	98.15	99.63
view-3	87.04	96.30

## Data Availability

The data are not publicly available due to privacy of volunteers.
